# Treatment with Lobeglitazone Attenuates Hepatic Steatosis in Diet-Induced Obese Mice

**DOI:** 10.1155/2018/4292509

**Published:** 2018-06-13

**Authors:** Sorim Choung, Kyong Hye Joung, Bo Ram You, Sang Ki Park, Hyun Jin Kim, Bon Jeong Ku

**Affiliations:** ^1^Department of Medical Science, College of Medicine, Chungnam National University, Daejeon 35015, Republic of Korea; ^2^Department of Internal Medicine, College of Medicine, Chungnam National University, Daejeon 35015, Republic of Korea

## Abstract

Nonalcoholic fatty liver disease (NAFLD) is strongly associated with insulin resistance. The peroxisome proliferator-activated receptor (PPAR) activators, thiazolidinediones, (TZDs), are insulin sensitizers used as a treatment for NAFLD. However, TZDs are a controversial treatment for NAFLD because of conflicting results regarding hepatic steatosis and fibrosis. To evaluate a possible effective drug for treatment of NAFLD, we investigated the effects of a newly developed TZD, lobeglitazone, with an emphasis on hepatic lipid metabolism. Lobeglitazone treatment for 4 weeks in high fat diet- (HFD-) induced obese mice (HL group) improved insulin resistance and glucose intolerance compared to HFD-induced obese mice (HU group). The gene levels related to hepatic gluconeogenesis also decreased after treatment by lobeglitazone. The livers of mice in the HL group showed histologically reduced lipid accumulation, with lowered total plasma cholesterol and triglyceride levels. In addition, the HL group significantly decreased the hepatic expression of genes associated with lipid synthesis, cholesterol biosynthesis, and lipid droplet development and increased the hepatic expression of genes associated with fatty acid *β*-oxidation, thus suggesting that lobeglitazone decreased hepatic steatosis and reversed hepatic lipid dysregulation. Livers with steatohepatitis contained increased levels of PPAR*γ* and phosphorylated PPAR*γ* at serine 273, leading to downregulation of expression of genes associated with insulin sensitivity. Notably, the treatment of lobeglitazone increased the protein levels of PPAR*α* and diminished levels of PPAR*γ* phosphorylated at serine 273, which were increased by a HFD, suggesting that induction of PPAR*α* and posttranslational modification of PPAR*γ* in livers by lobeglitazone might be an underlying mechanism of the improvement seen in NAFLD. Taken together, our data showed that lobeglitazone might be an effective treatment for NAFLD.

## 1. Introduction

Nonalcoholic fatty liver disease (NAFLD) is becoming a serious clinical problem because of an increased number of obese and overweight patients [1]. Although the number of NAFLD patients is growing rapidly, there is no optimal therapy for this disorder [2]. NAFLD is related to obesity, diabetes, hyperlipidemia, and a high fat diet (HFD), which are conditions linked with insulin resistance [3]. Insulin resistance causes an uncontrolled release of free fatty acids from adipose tissue and multiple alterations of fat metabolism in the liver [4]. NAFLD could therefore be induced by an imbalance caused by exacerbated hepatic lipid accumulation and ameliorated lipid build-up.

The peroxisome proliferator-activated receptor (PPAR) is a member of the nuclear receptor superfamily of ligand-inducible transcription factors, comprising PPAR*α*, PPAR*β*/*δ*, and PPAR*γ*. PPAR*γ*, which is a master regulator of gene expression in metabolic, inflammatory, and other pathways [5], improves insulin sensitivity through upregulation of glucose/lipid uptake and storage, mainly in adipose tissue [6]. The thiazolidinediones (TZDs) are synthetic activators of PPAR*γ* that induce insulin sensitization as a treatment for type 2 diabetes mellitus (T2DM). Rosiglitazone and pioglitazone, which are members of the TZD family, have been studied as the drug for the treatment of NAFLD. Many studies have suggested that rosiglitazone and pioglitazone indirectly improve NAFLD through enhancing fatty acid uptake and adiponectin secretion of the adipose tissue, which is the main organ that express the PPAR*γ* [7, 8]. It is still unclear whether the hepatic PPAR*γ* activation by TZD is main mechanism to directly improve hepatic steatosis.

Lobeglitazone, a dual activator of PPAR*α* and PPAR*γ*, has recently been approved in The Republic of Korea and is being used to treat T2DM following the completion of clinical trials [9]. Animal studies showed that lobeglitazone inhibited renal fibrosis through regulation of the TGF-*β*/Smad3 pathway [10] and ameliorated inflammation of white adipocytes [11]. T2DM patients treated with lobeglitazone showed improvements in hepatic steatosis, hyperglycemia, and insulin resistance [2, 12]. One study reported that lobeglitazone was mainly localized to the liver [13], suggesting that it could have potent effects for improving insulin sensitivity and lipid metabolism in the liver compared to other TZDs. However, there is no clear evidence that lobeglitazone improved NAFLD through direct effects on the liver.

In the present study, we therefore characterized the effects of lobeglitazone in an animal model of obesity-associated hepatic steatohepatitis, focusing on lipid metabolism in the liver.

## 2. Materials and Methods

### 2.1. Animals

Male C57BL/6J mice were purchased from Harlan (Indianapolis, IN, USA). A HFD (D12492) comprising 60% fat was purchased from Research Diets (New Brunswick, NJ, USA). The animals were maintained in a controlled environment (12 hours of light/12 hours of dark) with a humidity of 50%–60% and an ambient temperature of 22 ± 2°C. The 6-week-old male mice were fed a normal chow diet (NCD) or HFD for 8 consecutive weeks and then divided randomly into three groups: a group of mice fed the NCD without treatment (NU group), a group of mice fed the HFD without untreatment (HU group), and a group of mice fed an HFD with lobeglitazone treatment (Duvie, 5 mg/kg/day; oral gavage) for the final 4 weeks (HL group). All experimental procedures were conducted in accordance with the guidelines of the Institutional Animal Care and Use Committee, Chungnam National University School of Medicine, Daejeon, Republic of Korea.

### 2.2. Histological Analysis

In all groups, tissue samples were obtained from 18-week-old mice. Samples for light microscopy were fixed in 4% paraformaldehyde for 1 hour. Paraffin embedding, sectioning, hematoxylin and eosin (H&E), and Oil Red O staining were performed according to standard protocols. The Oil Red O stained sections were also quantified by digital image analysis (DIA) using image-dedicated software (ImageJ) [14].

### 2.3. Serum Biochemical Measurements

Blood was collected from the heart under general anesthesia. The samples were centrifuged at 5,000 rpm for 5 minutes and the supernatants collected. Serum insulin was measured using an ELISA kit (Alpco Diagnostics, Salem, NH, USA). Biochemical analyses, including determination of free fatty acids and total cholesterol, were performed using a Hitachi 7180 auto analyzer (Tokyo, Japan) and reagents (Wako Pure Chemical Industries, Osaka, Japan).

### 2.4. Intraperitoneal Glucose Tolerance Test (IPGTT) and Insulin Tolerance Test (ITT)

For the IPGTT, mice were fasted for 16 hours, and then 2 g/kg glucose was injected into the intraperitoneal cavity. Blood glucose levels were measured at 0, 15, 30, 60, 90, and 120 minutes using a glucometer (Accu-Chek; Roche, Basel, Switzerland). The ITT was performed by measuring blood glucose after 6 hours of fasting, followed by intraperitoneal injection of 0.75 U/kg insulin (Humalog; Eli Lilly, Indianapolis, IN, USA).

### 2.5. Western Blot Analysis

The livers from mice were lysed in RIPA buffer (30 mM Tris, pH 7.5, 150 mM sodium chloride, 1 mM phenylmethylsulfonyl fluoride, 1 mM sodium orthovanadate, 1% Nonidet P-40, 10% glycerol, containing phosphatase, and protease inhibitors). Western blot analyses were performed with 30–50 *μ*g protein from the tissue homogenate using commercially available antibodies to the following: antisterol responsive elementary binding protein (Srebp) 1 and Srebp2 (BD Biosciences, San Jose, CA, USA), fatty acid synthase (Fasn), PPAR*α* and PPAR*γ* (Cell Signaling Technology, Danvers, MA, USA), acid binding cassette A1 (ABCA1) (Millipore, Hayward, CA, USA), and phosphoPPAR*γ* (S273) (Bioss, Centennial, CO, USA). Secondary antibodies (goat anti-mouse and goat anti-rabbit) were obtained from Cell Signaling Technology.

### 2.6. Isolation of RNA and Analysis by Real-Time PCR

Total RNA was isolated using TRIzol reagent (Thermo Fisher Scientific, Scotts Valley, CA, USA), and cDNA was prepared from total RNA using M-MLV reverse transcription and oligo-dT primers (Invitrogen, Carlsbad, CA, USA). The resultant cDNA was amplified using Rotor-Gene™ 6000 real-time rotary analysis software (ver. 1.7; Corbett Life Science, Mortlake, Australia). Real-time PCR was performed in triplicate with individual time-matched, vehicle-treated, or control mice using QuantiTect™ SYBR® Green PCR Master Mix (Qiagen, San Diego, CA, USA). All quantitative calculations were performed using the ΔΔCT method.

### 2.7. Statistical Analysis

Statistical analyses were performed using Graph Prism 5 (GraphPad, La Jolla, CA, USA). Data are reported as means ± SEM. All data from animal studies were analyzed by two-way repeated-measures analysis of variance followed by Bonferroni correction for multiple comparisons, one-way analysis of variance followed by Tukey's post hoc test, or two-tailed Student's* t-*test. A p-value < 0.05 was considered statistically significant.

## 3. Results

### 3.1. Lobeglitazone Treatment Improves Insulin Resistance in HFD-Induced Obese Mice

**Figure 1:**
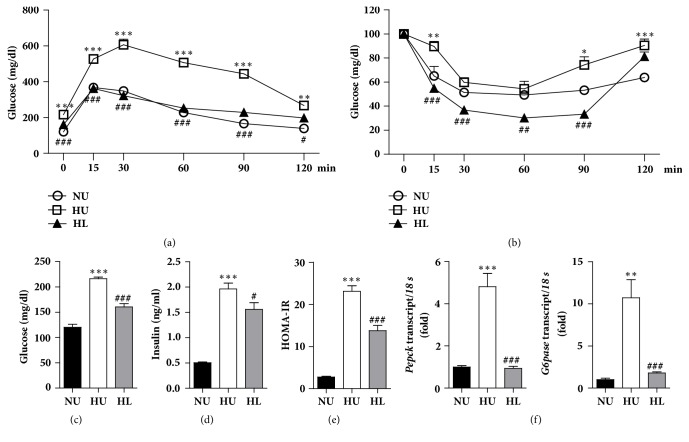
Lobeglitazone treatment improves systemic insulin resistance in high fat diet- (HFD-) fed mice. (a) The glucose tolerance test. Glucose (1 g/kg body weight) was intraperitoneally (i.p.) injected in overnight-fasted mice. (b) The insulin tolerance test. Blood glucose levels were measured at the indicated time points before and after i.p. injection of human regular insulin (0.75 U/kg) in mice fasted for 4 hours. (c) The fasting blood glucose level. (d) The fasting blood insulin level. (e) A homeostasis model for assessment of the insulin resistance index (HOMA-IR). (f) The gene expression levels of* PEPCK* and* G6Pase* related to gluconeogenesis were determined by qPCR in liver tissues. NU: normal chow diet- (NCD-) fed mice without treatment (NU, white circles and black bar), HU: HFD-fed mice without treatment (white squares and white bar), and HL: HFD-fed mice with lobeglitazone treatment (black triangles and gray bar). Data are expressed as means ± SEM. ^*∗*^*p* < 0.05, ^*∗∗*^*p* < 0.001, and ^*∗∗∗*^*p* < 0.0001, NU versus HU group. ^#^*p* < 0.05, ^##^*p* < 0.001, and ^###^*p* < 0.0001, HU versus. HL group.

After feeding of a HFD for 12 weeks, C57BL/6J mice showed a significant increase in body weight compared with mice fed a NCD (data not shown). We confirmed the effects of a HFD through which the HU group showed the glucose intolerance on the IPGTT (Figure 1(a)) and insulin resistance on the ITT (Figure 1(b)) compared with the NU group. The HL group significantly improved glucose tolerance and insulin sensitivity compared with the HU group (Figures 1(a) and 1(b)). Fasting plasma glucose and insulin levels were also markedly lower in the HL group compared with the HU group (Figures 1(c) and 1(d)), resulting in an improved homeostasis model assessment of insulin resistance (HOMA-IR) index in the HL group (Figure 1(e)). In addition, glucose-regulating enzymes in the liver, such as PEPCK and G6Pase, better suppressed the transcriptional levels of genes in the HL group when compared with the HU group (Figure 1(f)). These findings suggested that lobeglitazone efficiently improved hepatic insulin sensitivity in HFD-induced obese mice.

### 3.2. Lobeglitazone Treatment Prevents Hepatic Steatosis in HFD-Induced Obese Mice

**Figure 2:**
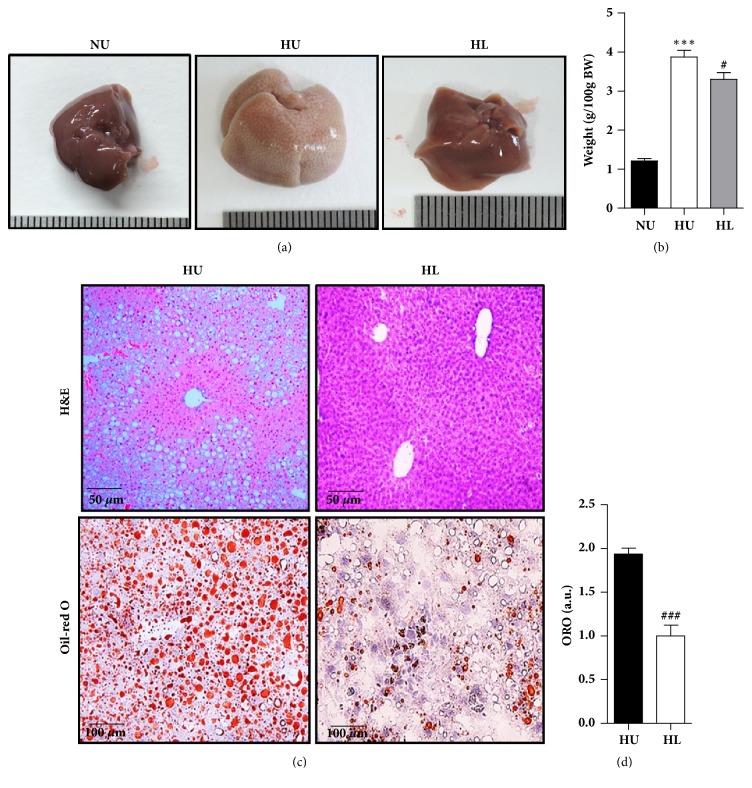
Lobeglitazone treatment ameliorates hepatic steatosis in HFD-fed mice. (a) Representative images of livers of NCD-fed mice without treatment (NU), HFD-fed mice without treatment (HU), and HFD-fed mice with lobeglitazone treatment (HL) after 12 weeks of a HFD. (b) Weight of the livers (g/100 g body weight) of NU (black bar), HU (white bar), and HL (black gray bar). (c) Representative images of hematoxylin and eosin staining and Oil Red O staining of liver sections of HFD-fed mice without treatment (HU) and HFD-fed mice with lobeglitazone treatment (HL). (d) Digital image analysis (DIA) quantification of Oil Red O stained sections. a.u., arbitrary units. Data are expressed as means ± SEM. ^*∗*^*p* < 0.05, ^*∗∗*^*p* < 0.001, and ^*∗∗∗*^*p* < 0.0001, NU versus. HU group. ^#^*p* < 0.05, ^##^*p* < 0.001, and ^###^*p* < 0.0001, HU versus. HL group.

Mice liver in the HU group were larger in size and have a lighter color, compared to the relative healthy red color of the mice liver in the HL group (Figure 2(a)). The weights of the livers in the HU group were drastically increased compared with those in the NU group (Figure 2(b)). The weights of the livers in the HL group were also significantly reduced compared to those in the HU group and the HL group exhibited markedly reduced hepatic accumulation of lipids, as assessed by H&E and Oil Red O staining (Figures 2(c) and 2(d)). These results suggested that lobeglitazone mitigated the hepatic steatosis induced by HFD.

### 3.3. Lobeglitazone Treatment Reduces the Serum Lipid Levels in HFD-Induced Obese Mice

**Figure 3:**
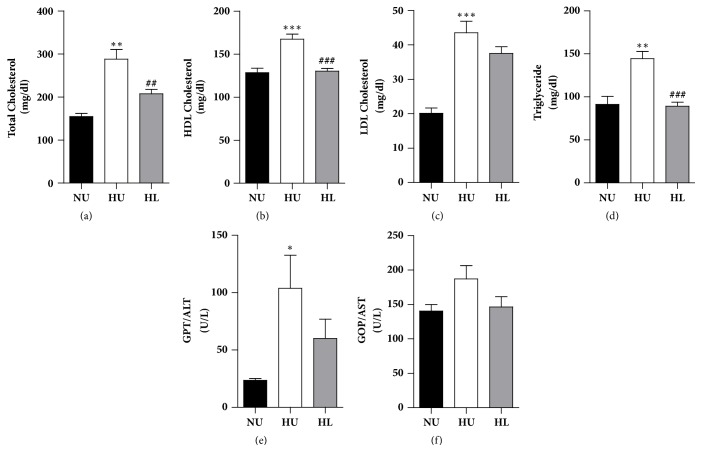
Lobeglitazone treatment reduces serum cholesterol and triglyceride (TG) levels in HFD-fed mice. Serum concentrations of lipid were measured after 12 weeks of a HFD or NCD. (a) Serum total cholesterol level. (b) Serum high density lipoprotein (HDL) cholesterol level. (c) Serum low density lipoprotein (LDL) cholesterol level. (d) Serum TG level. (e) Alanine aminotransferase (ALT) level. (f) Aspartate aminotransferase (ALP) level. NU: normal chow diet- (NCD-) fed mice without treatment (NU, black bar), HU: HFD-fed mice without treatment (white bar), and HL: HFD-fed mice with lobeglitazone treatment (gray bar). Data are expressed as means ± SEM. ^*∗*^*p* < 0.05, ^*∗∗*^*p* < 0.001, and ^*∗∗∗*^*p* < 0.0001, NU versus. HU group. ^#^*p* < 0.05, ^##^*p* < 0.001, and ^###^*p* < 0.0001, HL versus. HU group.

To identify whether lobeglitazone improved the serum lipid profile during histological mitigation of hepatic steatosis, we measured the serum levels of triglycerides (TGs) and cholesterol. Although there was no significant change in the levels of low density lipoprotein cholesterol (LDL-C) (Figure 3(c)), total and high density lipoprotein cholesterol (HDL-C) and TG levels were lower in the HL group than in the HU group (Figures 3(a), 3(b), and 3(d)). To confirm whether the HFD caused the steatohepatitis that was improved by lobeglitazone, we measured the serum levels of alanine aminotransferase (GPT/ALT) and aspartate aminotransferase (GOP/AST), which are hepatic injury markers. The ALT level, but not the AST level, was significantly higher in the HU group than in the NU group (Figures 3(e) and 3(f)), suggesting that the HFD resulted in hepatitis with hepatic lipid accumulation. However, ALT was not significantly lower in the HL group versus the HU group, although ALT was reduced in the HL group (Figures 3(e) and 3(f)) suggesting that lobeglitazone treatment for 4 weeks could not completely recover the hepatic injury caused by the HFD. Taken together, these results showed that lobeglitazone restored the serum lipid levels altered by a HFD.

### 3.4. Lobeglitazone Regulates Hepatic Lipid Metabolism in HFD-Fed Mice

**Figure 4:**
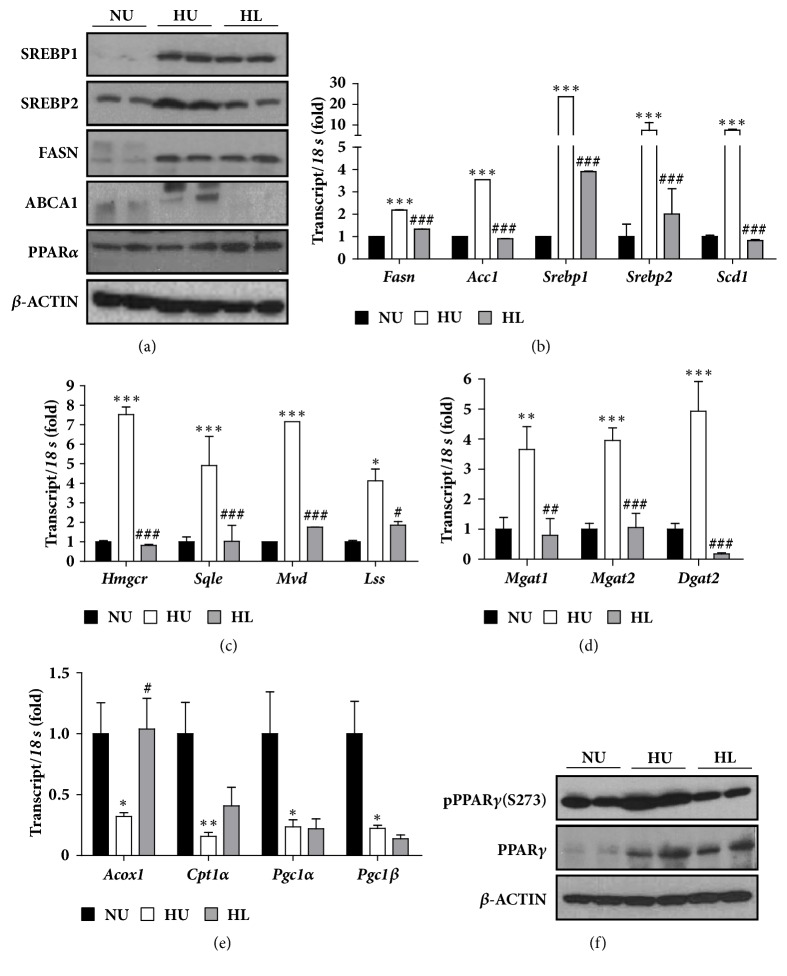
Lobeglitazone regulates hepatic lipid metabolism in HFD-fed mice. (a) Protein expression of lipid metabolism-related genes in liver tissues. ß-actin was included as a loading control. (b) Expression levels of de novo lipogenesis-related genes in liver tissues. (c) Expression levels of cholesterol biosynthesis-related genes in liver tissues. (d) Expression levels of lipid droplet development-related genes in liver tissues. (e) Expression levels of fatty acid *β*-oxidation related genes in liver tissues. (f) Protein expression of pPPAR*γ* (S273) and PPAR*γ* in liver tissues. ß-ACTIN was included as a loading control. NU: normal chow diet- (NCD-) fed mice without treatment (NU, black bar), HU: HFD-fed mice without treatment (white bar), and HL: HFD-fed mice with lobeglitazone treatment (gray bar). Data are expressed as means ± SEM. ^*∗*^*p* < 0.05, ^*∗∗*^*p* < 0.001, and ^*∗∗∗*^*p* < 0.0001, NU versus. HU group. ^#^*p* < 0.05, ^##^*p* < 0.001, and ^###^*p* < 0.0001, HU versus. HL group.

Because lobeglitazone ameliorated lipid accumulation in the liver and restored the serum lipid profile, we evaluated the levels of hepatic protein and gene expression associated with lipid metabolism. Using western blotting, the HL group decreased the protein levels of Srebp1, Srebp2, and ABCA for hepatic lipogenesis and increased the protein levels of PPAR*α*, which mainly included fatty acid *β*-oxidation and various lipid metabolisms of liver, compared with the HU group (Figure 4(a)). Transcriptional levels of key genes for de novo lipogenesis, such as acetyl-CoA carboxylase 1 (*Acc1*),* Srebp1*,* Srebp2,* and stearoyl-CoA desaturase 1 (*Scd1*), were significantly decreased in the HL group compared to the HU group (Figure 4(b)). Transcriptional levels of genes for cholesterol biosynthesis, such as HMG-CoA reductase* (Hmgcr),* squalene epoxidase* (Sqle),* mevalonate (diphospho) decarboxylase* (Mvd),* and lanosterol synthase* (Lss)*, were remarkably decreased in the HL group compared to the HU group (Figure 4(c)). The expression levels of genes for lipid droplet development, such as mannosyl (alpha-1,3-)-glycoprotein beta-1,2-N-acetylglucosaminyltransferase (*Mgat*) 1,* Mgat 2*, and diacylglycerol-o-acyltransferase 2 (*Dgat 2*), were also decreased in the HL group compared to the HU group (Figure 4(d)). However, transcriptional levels of acyl-CoA oxidase 1 (*Acox1*), known as a key gene for fatty acid *β*-oxidation, was increased in the HL group compared to the HU group (Figure 4(e)). These results showed that the beneficial effects of lobeglitazone on hepatic steatosis were associated with a decrease in hepatic lipid synthesis and an increase in fatty acid *β*-oxidation.

Recent studies suggested that TZD plays a role in posttranslational modification, as well as agonism, of PPAR*γ* [15]. We therefore evaluated the protein levels of PPAR*γ* and PPAR*γ* phosphorylated at S273 (pPPAR*γ* (S273)) in adipose and liver tissue using western blotting. Although the absolute protein level of PPAR*γ* and pPPAR*γ* (S273) could not compare the liver tissue with adipose tissue, the ratio of pPPAR*γ* (S273) to PPAR*γ* in the NU group was very high in liver than in adipose tissue (Figure 4(f) and Supplementary Figure 1). The protein level of PPAR*γ* in liver and adipose tissue were very high in the HU and HL group than in the NU group and did not show the difference between the HU and HL group (Figure 4(f) and Supplementary Figure 1). However, the protein level of pPPAR*γ* (S273) in liver and adipose tissue is significantly lower in the HL group than in the HU group (Figure 4(f) and Supplementary Figure 1). These results showed that lobeglitazone played a role in the posttranslational modification of PPAR*γ* not only in adipose tissue, but also in liver.

## 4. Discussion

Hepatic steatosis, the first stage of NAFLD, starts with an accumulation of TG in the cytoplasm of hepatocytes and may then progress to nonalcoholic steatohepatitis (NASH), fibrosis, and hepatocellular carcinoma [16, 17]. Although the pathogenic pathways associated with inflammation, apoptosis, and fibrosis have been studied by researchers as possible therapeutic targets [18], most drugs were not used for the treatment of NASH, either because of insufficient potency to inhibit the progression of NASH, or because of alternative pathways that retained the NASH phenotype. Current attempts to treat NAFLD have emphasized correcting insulin resistance, which is an almost constant finding in NAFLD [19]. PPAR*γ* activator TZD, which acts as a potent insulin sensitizer used for treatment of T2DM, has attempted as a treatment for NAFLD. Many studies showed that rosiglitazone and pioglitazone significantly improved hepatic steatosis [20, 21], and this effect of these drugs is largely explained by the secondary effect of improving the insulin sensitivity of adipose tissue, where PPAR*γ* level is mainly high [7, 8]. Recent studies have suggested that the structural differences among TZDs might not only result in increased PPAR*γ* expression, but also result in ligand-dependent posttranslational modifications of PPAR*γ* [15, 22]. Lobeglitazone contains a common TZD moiety, but with different side chains compared with rosiglitazone and pioglitazone [9, 13]. Therefore, we hypothesized that the novel TZD, lobeglitazone, could be used as a potent drug for the treatment of NAFLD in an HFD-induced obese mouse model.

Recently, T2DM patients treated with lobeglitazone not only improved their insulin sensitivity and glucose intolerance, but also showed an improvement in NASH [12]. Consistent with previous studies [9, 11], we confirmed that the HL group had decreased fasting blood glucose levels and enhanced insulin sensitivity when compared with the HU group (Figure 1). In addition, mice in the HL group had effectively increased weight of adipose tissue compared with the HU group (data not shown). It could indicate that lobeglitazone has worked appropriately as a PPAR*γ* activator which promotes the proliferation and differentiation of adipocytes [23]. Therefore, the effects of lobeglitazone in adipose tissue would play a role in improving the insulin sensitivity in the HL group. Mice in the HL group had decreased liver weight but did not have significantly lower levels of liver enzymes. Obesity involves the recruitment of macrophages according to lipid accumulation in the liver [24] and increases in levels of hepatic enzymes such as GPT/ALT and GOP/AST during the progression of an inflammatory reaction (i.e., NASH). In this study, although short-term lobeglitazone treatment for 4 weeks failed to reverse the inflammatory response in the HL group, the HL group not only showed improved lipid accumulation in the liver (Figure 2), but also significantly decreased total cholesterol and TG levels (Figure 3). Because the primary target site of lobeglitazone is the liver [13], lobeglitazone may first alter lipid metabolism rather than inflammatory pathway in the liver.

NAFLD can be induced by altering hepatic lipid metabolism, through enhancing hepatic lipid accumulation and attenuating the increase in serum lipid levels [25]. Because PPAR*α* is mainly expressed in liver tissue and play important roles in lipid uptake and storage of liver tissue, the regulation of hepatic PPAR*α* is necessary for the treatment of NAFLD. PPAR*α* plays an important role in lipid catabolism through induction of mitochondrial fatty acid *β*-oxidation in liver tissue [26]. A recent study reported that hepatic PPAR*α* played a central role in the clearance of free fatty acids released from adipocytes, which are the major source of lipids in NAFLD [27]. In this study, PPAR*α* levels in the HL group increased in the liver with transcriptional induction of genes involving fatty acid *β*-oxidation, as well as transcriptional inhibition of genes involved in lipid synthesis, cholesterol biosynthesis, and lipid droplet development (Figure 4). Therefore, the effects of lobeglitazone on NAFLD could be due more to the effect on PPAR*α* than on rosiglitazone and pioglitazone.

PPAR*γ* was generally lower levels in liver tissue in adipose tissue and could have less important roles than PPAR*α* for lipid metabolism of liver. However, in this study, HFD in mice induced the protein levels of PPAR*γ* and phosphorylation of PPAR*γ* at S273 (pPPAR*γ* (S273)), not only in adipose tissue but also in liver tissue (Figure 4(f) and Supplementary Figure 1). Treatment of lobeglitazone lowered the pPPAR*γ* (S273) in adipose and liver tissue (Figure 4 and Supplementary Figure 1). Cdk5-mediated phosphorylation of PPAR*γ* at S273 induced by HFD has been linked to insulin resistance [15]. One study also reported that inhibition of pPPAR*γ* (S273) ameliorated hepatic steatosis [28]. Therefore, the improvement of hepatic steatosis in the HL group might have been associated with a decrease in this phosphorylation although we did not confirm whether pPPAR*γ* (S273) in the liver altered gene expression.

In conclusion, we showed that lobeglitazone conferred potentially beneficial effects on insulin sensitivity and hepatic steatosis through improvement of lipid metabolism via altering the expression of target genes involved in these pathways. The changes in gene expression may be due to the induction of PPAR*α* and inhibition of pPPAR*γ* phosphorylation at S273. This study therefore suggests an important role for lobeglitazone in modulating hepatic steatosis, as well as suggesting a novel therapy for the treatment of NAFLD.

## Supplementary Materials

Supplementary Materials Supplementary Figure 1: protein expression of pPPAR*γ* (S273) and PPAR*γ* in adipose tissues. ß-ACTIN was included as a loading control.
