# Supplementary figures and images for: Treatment with Lobeglitazone Attenuates Hepatic Steatosis in Diet-Induced Obese Mice
Source: PPAR Res. 2018 Jun 13;2018:4292509. doi: 10.1155/2018/4292509 (PMC6020545; doi:10.1155/2018/4292509)

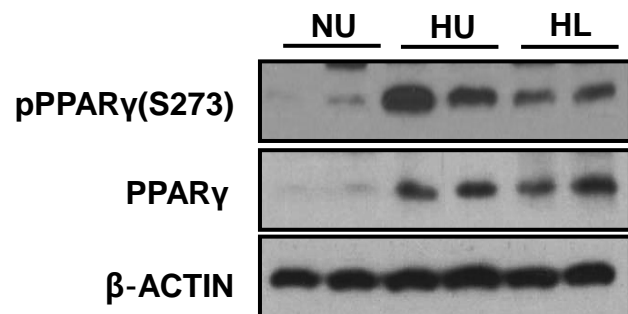

Supplement: Supplementary Materials — Supplementary Figure 1: protein expression of pPPARγ (S273) and PPARγ in adipose tissues. ß-ACTIN was included as a loading control. [file 4292509.f1.pdf]
